# Medical Imaging: From Roentgen to the Digital Revolution, and Beyond

**DOI:** 10.5041/RMMJ.10355

**Published:** 2018-10-04

**Authors:** Eyal Bercovich, Marcia C. Javitt

**Affiliations:** 1 Department of Medical Imaging, Rambam Health Care Campus, Haifa, Israel; 2 The Ruth & Bruce Rappaport Faculty of Medicine, Technion–Israel Institute of Technology, Haifa, Israel

**Keywords:** Machine learning, medical imaging, radiology, radiomics, theranostics

## Abstract

Today medical imaging is an essential component of the entire health-care continuum, from wellness and screening, to early diagnosis, treatment selection, and follow-up. Patient triage in both acute care and chronic disease, imaging-guided interventions, and optimization of treatment planning are now integrated into routine clinical practice in all subspecialties. This paper provides a brief review of major milestones in medical imaging from its inception to date, with a few considerations regarding future directions in this important field.

## THE BEGINNING

Until November 8, 1895, physicians had no access to pictures of what was happening inside a patient’s body. On that fateful day, Wilhelm Conrad Roentgen discovered a “new kind of ray” that he called “X-rays” while experimenting with a Crookes cathode ray tube. Because X-ray absorption is dependent on density, X-ray absorption by bones, but less so by soft tissues and even less by fatty tissue, created a clear picture. The first and most famous X-ray image in history was of the hand of Roentgen’s wife Bertha. Roentgen’s discovery set in motion a revolution in medical diagnosis that continues to this day.^1^ Because X-ray absorption is dependent on density, a clear picture was created by marked X-ray absorption by bones, but less so by soft tissues and even less by fatty tissue. Using modern jargon, Roentgen’s discovery went “viral” within days. The new technology was received by laymen and the medical establishment alike with amazement.^2^

The Crookes tube used by Roentgen was quickly replicated in other laboratories around the world. In no time the device was used to examine bone fractures. In the first year after the discovery of X-rays, 1,044 scientific articles were published on the subject.^3^ Six years later, in 1901, Roentgen was awarded the Nobel Prize in Physics.^4^ As radiographs became an integral part of medical diagnosis, new applications and information about disease representation quickly amassed.

In the ensuing decades, radiography became essential for medical diagnoses. Military medicine adopted the technology to evaluate battlefield injuries as early as World War I. Soon after winning a Nobel Prize for her research, Marie Curie drove a truck with an X-ray machine to the battle front in France.^5^ Shortly thereafter, fluoroscopy was used early in the development of angiography.^6^

## FROM THE SHADOWS NEW MODALITIES ARISE

For decades radiographs were the only form of medical imaging, but human curiosity and the insatiable appetite to improve medical imaging grew. New tools were rolled out, and two-dimensional flat images gave way to three-dimensional imaging, four-dimensional imaging (three-dimensional imaging in real time), functional imaging, and molecular imaging.

### Computed Tomography

In the beginning of the twentieth century, Alessandro Vallebona, an Italian radiologist, invented conventional tomography. Conventional tomography is created by simultaneously moving the X-ray source and X-ray detector in tandem so as to keep an object of interest in the center point of the scan plane and blur out all other objects. Conventional tomography evolved, but it was still ineffective for imaging soft tissues and large areas of the body.^7^

Seventy-six years after the birth of medical imaging, computed tomography (CT) was developed using radiographic projections from multiple angles and then building a two-dimensional image with a mathematical model that incorporated all of the projected data. In 1967, Sir Godfrey Hounsfield invented the first CT scanner at EMI research laboratories. The first live patient was scanned on October 1, 1971. Alan M. Cormack (who created the early mathematical models used in CT) and Hounsfield jointly received a Nobel Prize in 1979 for development of “computer assisted tomography.”^4^

Computed tomography is one of most important medical innovations in human history. Images display soft tissue contrasted with anatomic detail, facilitating unprecedented diagnostic accuracy. Two-dimensional, cross-sectional images are created that can be arranged in space to generate a volume. By the early 1980s, more than three million CT studies had been performed. Presently that number has grown to well over 100 million CT studies annually,^8^ with CT becoming the modern doctor’s “truth machine.”

Many technological advancements were made in CT, with improvements in scan speed, smaller slice thickness, decreased radiation dose, and better image quality. Twenty-five years ago, a typical CT study could take dozens of minutes depending on the scanned volume and exact machine, whereas nowadays CT studies can be performed in a fraction of a second, covering large areas of the body, even using more than one scan energy. The CT slices in modern machines can be as thin as fractions of a millimeter.^7^ New image reconstruction techniques and more efficient, larger detector materials have decreased radiation dosages by more than half,^9^ while continuously improving image quality.

**Figure 1:**
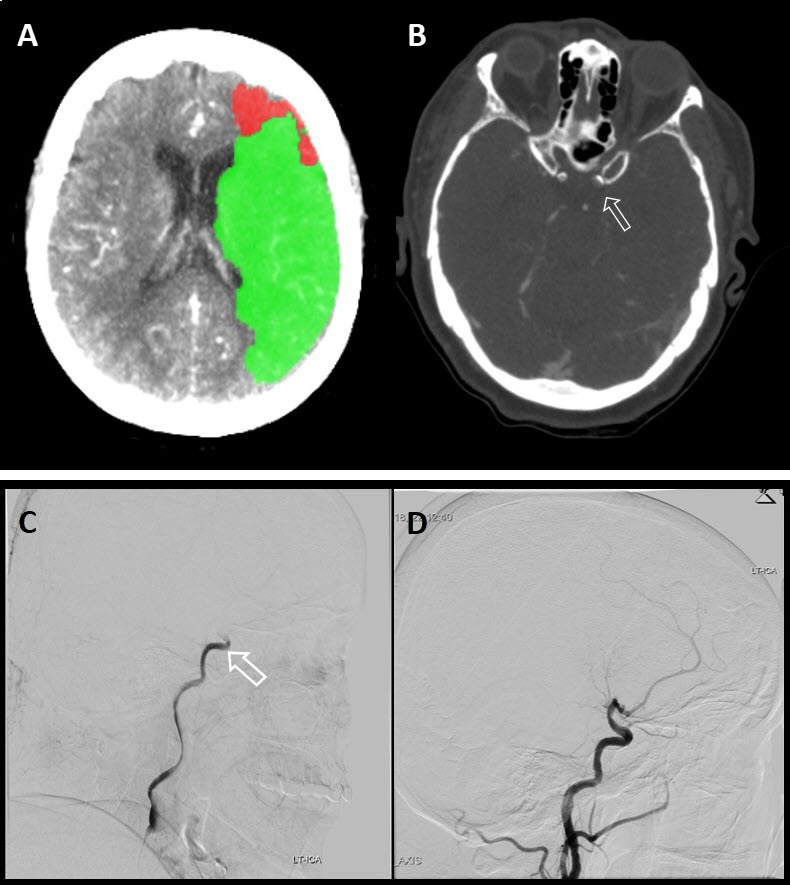
CT Brain Perfusion, CTA, Brain Angiography A: A CT perfusion algorithm calculation showing decreased perfusion in a brain area consistent with left middle cerebral artery (MCA) infarct, the green area representing penumbra. B: CT angiography showing the occluded intracranial left carotid artery with a filling defect (arrow) indicating that the artery is occluded. C: Cerebral catheter angiography showing the occluded distal left carotid artery (arrow). D: Cerebral catheter angiography after successful retrieval of the clot using a stent retriever.

New applications, such as CT perfusion for detection and quantification of cerebral stroke, soon followed. Recently, the therapeutic window (i.e. the timeframe within which treatment can be initiated as measured from the onset of symptoms) significantly increased thanks to the accuracy of perfusion CT, affording many more patients the chance for effective embolectomy. If CT perfusion reveals a sufficient penumbra, neuro-interventional therapy may be attempted up to 24 hours after the onset of symptoms.^10^ Together with CT angiography, this new approach has revolutionized the world of stroke therapy (Figure 1).

Another frontier is cardiac CT with coronary CT angiography (CTA). In the setting of low- to moderate-risk chest pain, coronary CTA is a non-invasive method of evaluation with high sensitivity and specificity.^11^

Future directions include radiation dose reduction and spectral (multi-energy) CT. Spectral CT uses a single acquisition performed at multiple energies to extract more information about tissue differentiation based on the way different photon energies are absorbed in different tissues. Clearly, CT will continue to bring new insights into human diseases.

### Magnetic Resonance Imaging

The history of the development of magnetic resonance imaging (MRI) is complex and involves many contributions from clinical, scientific, and technical fields. Two scientists, Felix Bloch and Edward Purcell, simultaneously and independently developed the concept of nuclear magnetic resonance (NMR), for which they were jointly awarded the Nobel Prize in Physics in 1952.^4^ In 1973, Paul Lauterbur published the first NMR images,^12^ and he later received the 2003 Nobel Prize in Physiology and Medicine.^4^

Magnetic resonance imaging is based on different physical principles than CT. A powerful magnet is used to produce a very strong fixed magnetic field around the patient, and radiofrequency pulses are used to excite protons within the body. As the excited protons relax back to a resting state, they return signals that are captured and mapped into an image.

Today, MRI is used in virtually every medical subspecialty. Its superior soft tissue contrast and anatomic detail make it the crown jewel of medical imaging. It has become essential in oncology, where scanning is routinely utilized for preoperative staging and to determine the extent of disease throughout the body. Whole-body MRI is now a reality, especially when combined with functional nuclear medicine imaging, i.e. positron emission tomogramphy MRI (PET-MRI); the surface has barely been scratched for what is possible via this modality.

Cardiac MRI is increasingly used for conditions such as cardiomyopathy or acute myocardial infarction. Not only morphology, but also cardiac function can be assessed with cine images of ventricular wall motion, valvular function, and T1 mapping of cardiac muscle tissue fibrosis.^13^–^15^

**Figure 2:**
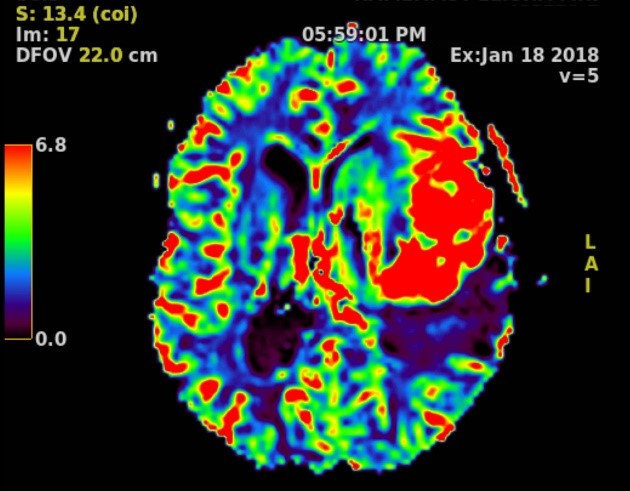
Magnetic Resonance Perfusion Brain perfusion MR sequence demonstrating markedly increased cerebral blood volume (red) in the left frontal lobe in a space-occupying lesion. An area of low blood volume is seen posteriorly representing the edema around the lesion (purple).

Functional MRI (fMRI) is used in clinical imaging of central nervous system diseases (e.g. vascular mapping for diagnosis and grading of gliomas) (Figure 2).^15^ Because fMRI measures brain activity by detecting changes associated with blood flow, it can be used in surgical planning. Robust research initiatives with fMRI and diffusion tensor imaging to study brain and spine nerve fiber tracts are underway.^16^,^17^ Magnetic resonance spectroscopy is used to measure the relative concentration of different endogenous metabolites in tissues.^18^

### Ultrasound

Ultrasound (US) is not one technology but many, with an intricate history involving many brilliant scientists and physicians since its inception in the twentieth century. The backstory of diagnostic ultrasonography began with the study of bats in the late 1700s by Lazzaro Spallanzani. Spallanzani tried to understand how bats fly at night and hypothesized that bats relied on sound to navigate.^19^ In 1826, Jean-Daniel Colladon calculated the speed of sound in water estimating close to the currently known value of 1,482 m/s.^20^ The Doppler effect, first postulated in 1842 by Christian Doppler, is an essential part of almost every US study and forms the basis of US evaluation of blood flow. Next the Curie brothers discovered piezoelectricity. Piezoelectric crystals vibrate when subjected to an alternating current. This is the basis for the development of US transducers.^21^

Interestingly the use of US in medicine started with therapeutic applications, not with imaging. The potentially destructive power of a focused US beam was recognized as a valuable therapeutic tool in the second decade of the twentieth century as a surrogate for neurosurgery, rehabilitation, and treatment of rheumatoid arthritis. In the 1940s, US for medical imaging was first investigated by a physician named Karl Theo Dussik. Dussik attempted to locate brain tumors and identify the cerebral ventricles. His initial experiments were reported in 1942.^20^,^22^ In 1958, Ian Donald, a gynecologist, first used US to study the unborn fetus, uterus, and pelvis.^23^

Ultrasound uses high-frequency sound waves, above the range of human hearing, which are transmitted into the body. The echoes reflected are mapped into an image based on the amplitude and time delay of the returned signals. This modality has advantages over CT. Because US has no ionizing radiation, is inexpensive, is widely available, and is performed face-to-face with patients, it is particularly important in pediatric imaging. Although conventional US is operator-dependent, new three-dimensional US is less so.

**Figure 3:**
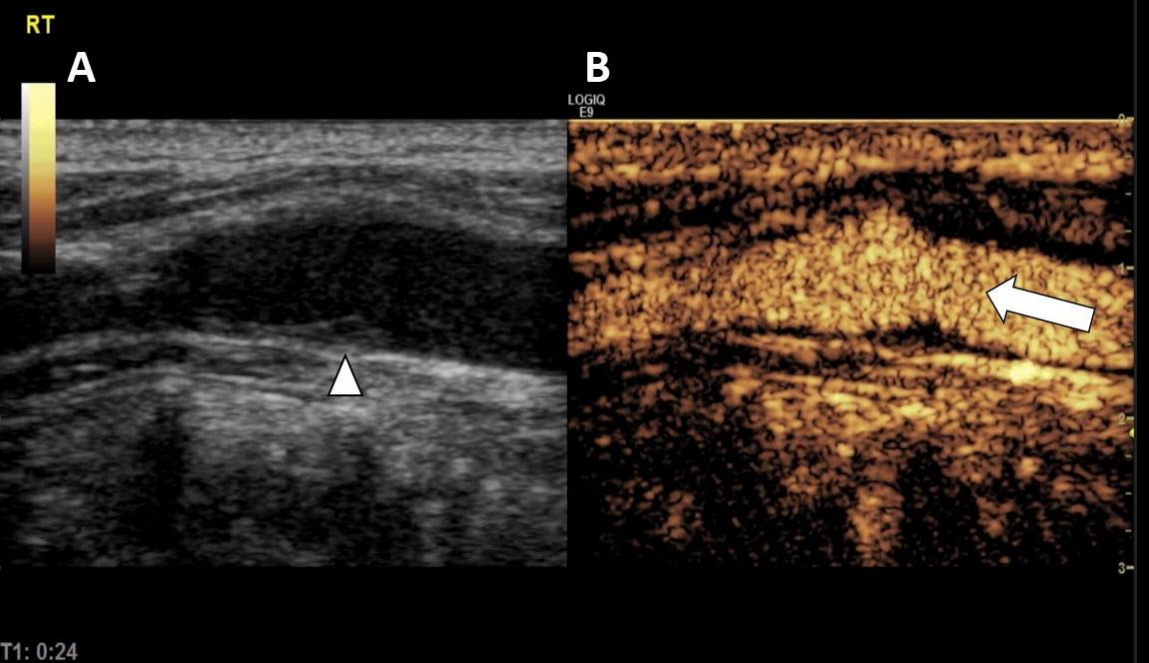
Contrast-enhanced Ultrasound (CEUS) Side by side: ultrasound grayscale image (A) and contrast-enhanced ultrasound image (B) of the carotid artery demonstrating the differentiation of the arterial wall atherosclerotic changes (arrow head) and blood flow in the vessel lumen (arrow).

A significant US advance was the development of US contrast materials comprising microbubbles that enhance the echo signal reflected from the imaged tissue. Contrast-enhanced US (CEUS) not only improves direct visualization of blood vessels, but also permits tissue characterization in solid organs. Using dynamic enhanced scans, lesion perfusion combined with the superior resolution of modern US can reveal microvasculature and may help unlock the mysteries of tumor angiogenesis in micron-sized abnormal blood vessels (Figure 3).^24^

Ultrasound elastography, a relatively new technique, provides semi-quantitative and quantitative measurements of tissue stiffness. Because tumor tends to be stiffer than surrounding normal tissue, elastography can assist with both lesion detection and characterization, and has been used in breast, thyroid, and liver imaging.

**Figure 4:**
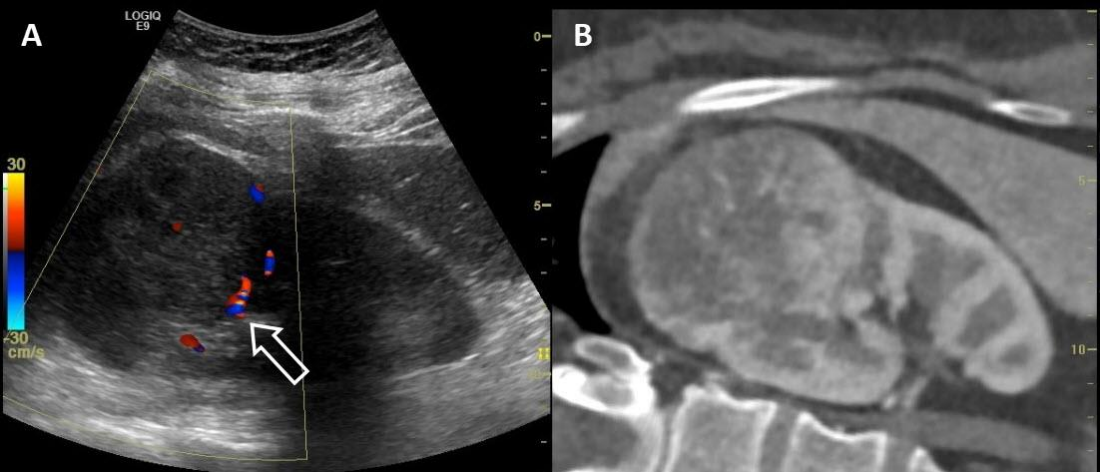
Ultrasound Computed Tomography (US-CT) Fusion US-CT fusion side by side: US color doppler (A) and CT images (B) of a US-CT fusion study. Longitudinal view of the kidney showing a space-occupying lesion in the upper pole of the kidney. Fusion imaging allows real-time assessment of tumor blood flow (arrow).

Another promising development is US fusion, where other modalities such as CT or MRI are combined with US. The different scans are spatially co-registered using the patient’s own anatomic landmarks. During real-time scanning, the US transducer can be moved using electromagnetic sensors and a field generator that enables comparison of lesions depicted on several modalities. It also permits navigation using real-time US for focal therapy such as for prostate cancer, based on the previously detected lesions in other modalities (Figure 4).^25^,^26^

### Nuclear Imaging and Hybrid Scanners

Nuclear medicine uses radioactive tracers to create functional imaging. Unique sensors detect the radioactive emission of these tracers. Single-photon emission computed tomography (SPECT) and positron emission tomography (PET) both use co-registration of a morphological scan (commonly CT) with a functional nuclear medicine scan.

The SPECT scans measure gamma rays, while PET scans use a tracer that accumulates in body tissues and is metabolized, after which positrons are emitted. When positrons encounter electrons, they annihilate each other, emitting a pair of gamma rays simultaneously. Special detectors register the simultaneous arrival of the pair of gamma rays and the number of such arrivals. The data are used to map metabolic activity in the organs of the body. James Robertson built the first single-plane PET scanner at the Brookhaven National Laboratory in 1961.

Functional and morphological co-registration (i.e. the first hybrid design) was first proposed for a PET-CT scanner by David Townsend and Ronald Nutt. A prototype hybrid clinical PET-CT scanner in a single gantry was first used clinically in 1998.^27^ Functional metabolic imaging is now routinely combined with high spatial resolution scans in clinical practice using not only PET-CT but also PET-MRI, and more.

### Interventional Medical Imaging

One important early development in interventional imaging was catheter angiography. In 1927, Egas Moniz, a neurologist from Portugal, performed the first cerebral angiography, for which he was awarded the Nobel Prize in Medicine in 1949.^28^ In 1929, the first cardiac angiography was performed by Werner Forssmann in Germany when he inserted a catheter into his own arm vein and passed a catheter into his heart. He shared the 1956 Nobel Prize in Medicine with Andre F. Cournand and Dickinson W. Richards for pioneering cardiac catheterization.^29^ Recent breakthroughs in catheter angiography for diagnosis and treatment of cardiovascular emergencies include removal of brain clot using new generations of retrievable stents, occlusion of active arterial hemorrhage in trauma patients, and in selective tumor ablation.

Other image-guided therapies have rapidly gone from the bench to the bedside in the last two decades. Image-guided biopsy is used daily in almost every organ including thyroid, lung, liver, breast, and bone. Minimally invasive procedures for abscess drainage and directed tissue ablation using real-time imaging guidance are in common practice. Tissue ablation may be accomplished using radiofrequency ablation, cryoablation, or stereotactic laser ablation.

**Figure 5:**
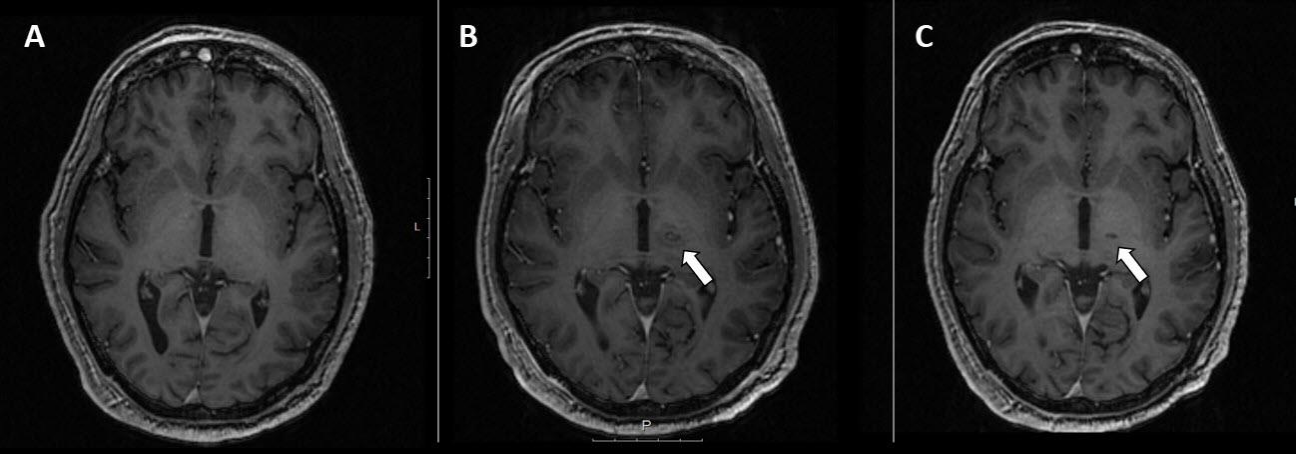
Magnetic Resonance-guided Focused Ultrasound (MRGFUS) Axial T1-weighted post-contrast MR images (A) before MR-guided focused ultrasound (MRGFUS) treatment. B: One day after MRGFUS to the right basal ganglia, showing the ablated area with surrounding edema (arrow). C: One year after treatment, showing a small area of encephalomalacia (arrow).

Another example, high-intensity focused ultrasound (HIFU), appeared in the early history of US. First proposed by Lynn et al. in 1942,^20^,^21^ HIFU guided by real-time imaging uses an external beam of therapeutic US to focally heat and ablate targeted tissues without damaging the surrounding normal tissue. The result of HIFU is irreversible coagulation necrosis and mechanical damage through cavitation. For example, MR-guided focused ultrasound (MRGFUS) has been successfully used to treat medication-resistant Parkinson’s disease.^30^–^32^ This technology is also used for many other conditions and locations ranging from movement disorders requiring ablation of deep brain nuclei (Figure 5), to ablation of uterine fibroids, prostate cancer, and bone metastases.

## EVOLUTION AND DIGITAL REVOLUTION

The success story of medical imaging during the last few decades is not only about technological advances but also the digital revolution. Digital images on workstations now replace films, permitting multiplanar image reconstruction.

Modern radiology is actually “filmless” but image-rich with digital data. Radiologists can be located far from the patient, remotely interpreting studies in real time, or long after the patient has left the point of care. Digital images have empowered teleradiology with remote image interpretation for primary or secondary readings.

Computer software and hardware advances have improved workstations used for medical imaging interpretation, giving radiologists the ability to process huge amounts of data, compare prior studies, and create multiplanar and three-dimensional image reconstruction. The picture archiving and communication systems (PACS) industry has developed in parallel with the needs of medical imaging to manage and store digital data.^33^,^34^ Multiple vendors have developed different competing systems to provide the most advanced image processing capabilities, all aimed at making the radiologists more efficient and more accurate.

The electronic health record is another important result of the digital revolution. Radiologists can access medical history, lab results, clinical notes, and comprehensive health information while they are protocoling or reading an imaging study. The radiology information system and hospital information system can now be integrated with coding and billing, workflow dashboards, and computerized order entry with decision support tools in modern hospitals. The radiologist has become an integrator of knowledge. Past medical history, previous studies, and lab results often influence the differential diagnosis offered by the reading radiologist.

Though often overlooked, another consequence of the digital revolution is the transformation of education in medical imaging. In the past, films were stored and teaching files created in an analogue space-occupying film library. Today, case-based teaching can be done using a simple search in the digital PACS system or via the Internet. This, together with current trends in social media and open collaboration, has created unprecedented access to a vast knowledge base for teaching and creation of enduring materials.

## MEDICAL IMAGING’S CHALLENGES

Increasing health-care costs have been linked in part to overutilization of imaging, with a continuous increase in the number of imaging studies. The fast-paced growth in the imaging knowledge base combined with the use of the electronic health records has led to a more than 10-fold increase in the amount of data managed daily by imaging specialists. The human limit for interpretation has been reached, resulting in an increased risk of medical errors,^35^ as well as increased risk of fatigue and burn-out for radiologists. The overwhelming increase in data acquired has resulted in some useful data created on scans, but the data have been neither extracted nor utilized.

Increased study volumes occur not only because of justified changes such as new clinical indications for scans, but also because physical examination is a dying art. While it is true that preventive medicine results in unnecessary tests, there is also an increasing reliance on laboratory and imaging testing as surrogates for clinical excellence. The use of imaging studies has grown faster than any other medical service.^36^

With the increasing volume of CT scans, there is an increasing population radiation burden. Today it is a well-known fact that exposure to ionizing radiation from CT and nuclear medicine results in an increased malignancy risk.^37^

## FUTURE DIRECTIONS—THE MACHINES ARE COMING

The challenges and opportunities for medical imaging will be defined by the way radiologists work and how they generate their work products, i.e. the radiology reports. Computers are contributing more and more to optimizing these end-products.

Machine learning (ML) used in facial recognition, self-driving cars, and voice recognition has exciting potential for medical applications. A form of artificial intelligence (AI), ML facilitates computer learning without explicit programming. The ML algorithms train computers using known datasets to build models for future predictions.

Computer-aided diagnosis (CAD) is one application for ML. Unlike conventional CAD, in which an algorithm was programmed to find known features, ML-based CAD uses algorithms to produce their own mathematical models for detection. For example, both algorithms may be used to identify birds. One algorithm is taught verbally with the definition of the features of a bird (e.g. “a flying animal with wings and feathers”). The other algorithm employs several bird pictures that reinforce, “this is a bird.” The second method is supervised machine learning that does not require the programmer to define a bird or its features.

**Figure 6:**
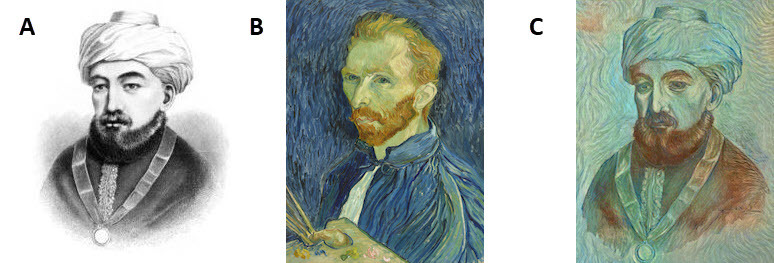
Transformation of the Original Painting of Moses Maimonides into the style of Vincent van Gogh A: The original painting. B: Painting used by a deep learning algorithm to transform the original (*Self-Portrait*, by Vincent van Gogh, 1889/Shutterstock.com). C: Final transformation of the original painting.

Current applications for ML include lesion detection such as that of brain hemorrhage, or lung nodules.^38^–^41^ Other algorithms are used to “teach” computers to transform new images to a preset pattern (Figure 6).^42^ Initial examples came from the world of fine art. Similar promising improvements can transform US image styles to a more uniform easily readable format.^43^

## WILL RADIOLOGISTS BE REPLACED BY MACHINES?

It is highly unlikely that machines will replace radiologists, in part because of the significant limitations of AI. In particular: (1) algorithms require very precise training and huge accurately labeled datasets; and (2) algorithms can answer very specific clinical questions, but there is selection bias.

Algorithms are often “black boxes;” understanding the process of decision-making might not be possible, which makes validation and regulation difficult.

Nevertheless, radiologists are desperate for help, and ML may provide this. Potentially, radiologists could collaborate with ML developers to find ways for ML to enhance productivity and improve accuracy. To that end, “augmented intelligence” might be a preferable term to AI.

The next stages must include the identification of AI performance metrics and establishment of standards that are sorely lacking, a shift from algorithms to data-driven analytics, more proposals that serve the end users’ immediate needs, practical workflow solutions, and training enhancements.

## RADIOMICS

Radiomics is the use of complex ML data characterization algorithms to extract quantitative features from medical imaging studies. It has the potential to discover completely new imaging information, mainly because of the algorithm’s ability to analyze and compare tens of thousands of studies. Radiomics truly leverages the advantage the technology has over human readers. Radiologists cannot analyze enormous datasets, but this can rapidly be accomplished using computer algorithms, rendering the contribution of this technology invaluable with untold new applications.

### Clinical Decision Support

The use of computerized order entry and decision support at the point of care has been developed and deployed in many countries. Using these systems, during the process of ordering medical imaging studies, the primary care physicians and ordering providers are directed to order the most appropriate study given a certain clinical scenario. The main goal of this process is to safely and efficiently acquire imaging data for rapid diagnosis while minimizing cost.

The American College of Radiology (ACR) has issued Appropriateness Criteria® (AC) with imaging guidelines that rank the most appropriate test for multiple clinical conditions,^44^ assist radiologists, and empower them to become better consultants for study selection. In the aggregate, these guidelines form the backbone of most modern clinical decision support tools. Clinical decision support software is being deployed around the world as a mechanical gatekeeper.

In the United States, the Protecting Access to Medicare Act (PAMA) legislation will soon require a referring provider to consult appropriate use criteria prior to ordering CT, MR, and nuclear medicine studies for Medicare patients.^45^ Although the aim is to reduce health-care costs, there are two secondary benefits: reduction of patient radiation exposure and reducing unnecessary testing by choosing the most appropriate study first. Seminal recent work, such as the Image Wisely campaign (www.imagewisely.org), is designed to educate primary care physicians about when it is appropriate to order studies and which studies to order with an emphasis on reducing radiation exposure.

### Point of Care and Home Care Medical Imaging

Medical imaging devices are being placed into the hands of non-radiologists across all subspecialties. There is controversy about the accuracy and advisability of interpretations from some inexperienced providers from primary care, emergency rooms, intensive care units, etc. However, if managed correctly, the widespread adoption of point of care US (POCUS), for example, can be a huge help to radiologists, first responders, primary care physicians, and patients. The key to success is education and collaboration with medical imagers.

Use of POCUS plays a salient role in both internal medicine and pediatrics as it is rapidly replacing the conventional stethoscope. Given the development of compact, ultra-mobile, inexpensive, handheld US devices, POCUS brings the device to the patient, rather than the other way around. Radiologists and primary care physicians must build a knowledge transfer platform wherein radiologists educate non-radiologists. This could accelerate diagnosis and treatment, thereby improving workflow. At the core of this system is evidence-based and data-driven medical decision-making.

### The Quest for the “Super” Modality

Streamlining the amount of data while increasing its value is a goal for the future of medical imaging. Creation of a “super modality” may occur through technical innovations such as multimodality fusion. Multimodality fusion with co-registration is now feasible, having been performed by marrying real-time US with CT, PET-CT, and multi-energy CT. The use of perfusion or functional sequences in MRI can be superimposed on conventional morphological sequences. The benefits of combined multimodality scans include increased diagnostic accuracy, reduced data burden on radiologists, lowered medical imaging costs, improved patient safety due to reduced radiation exposure, and shortened time-to-diagnosis. To better manage resources, it is likely that selective data acquisition with leaner and more focused imaging will continue to evolve.

### Theranostics

The concept of *theranostics* (i.e. synthesis of “therapeutics” and “diagnostics”^46^) is the creation of a “find it and fix it” solution using a single combined technology that both localizes and treats neoplasms. An early example of theranostics is the use of radioiodine to treat thyroid disease. “Super modalities” that identify targets using radiomics and treat using new minimally invasive methods are under development. New theranostic treatments exist for prostate cancer using prostate-specific membrane antigen (PSMA)-directed radioligand therapy (PRLT) and neuroendocrine tumors with peptide receptor radionuclide therapy (PRRT).^47^,^48^

The notion of finding imaging biomarkers of disease along with prediction of response to therapy is important because of the heterogeneity of tumors with their hosts. The magnitude of this variability is not always appreciated, given the small tissue volumes sampled with percutaneous biopsies.^46^,^48^,^49^

Theranostics can be used to identify the patients who would benefit from a customized specific therapy. Medical imaging with AI is a critical tool in this enterprise. Radiomics data acquired using AI can reveal heretofore unexplored imaging data that can be critical to future medical decision-making.

## SUMMARY

Advancements in image acquisition, analysis, transfer, and storage have put medical imaging at the forefront of medical diagnosis. Patient triage, treatment planning and optimization, and real-time image-guided interventions are now integrated into routine clinical practice in most disciplines.

In this review we have described medical imaging from its birth in 1895 to date. The challenges and the exciting new opportunities show promise for a bright future ahead. Advances in medical imaging will transform medicine for the masses into precision medicine with personalized care.

## Abbreviations

ACR: American College of Radiology
AC: Appropriateness Criteria
AI: artificial intelligence
CAD: computer-aided diagnosis
CEUS: contrast-enhanced ultrasound
CT: computed tomography
CTA: coronary CT angiography
fMRI: functional magnetic resonance imaging
HIFU: high-intensity focused ultrasound
ML: machine learning
MRGFUS: MR-guided focused ultrasound
MRI: magnetic resonance imaging
NMR: nuclear magnetic resonance
PACS: picture archiving and communication systems
PAMA: Protecting Access to Medicare Act
PET: positron emission tomography
PET-CT: positron emission tomography–computed tomography
PET-MRI: positron emission tomography magnetic resonance imaging
POCUS: point of care US
SPECT: single-photon emission computed tomography
US: ultrasound
